# An Assessment of Cold Hardiness and Biochemical Adaptations for Cold Tolerance Among Different Geographic Populations of the *Bactrocera dorsalis* (Diptera: Tephritidae) in China

**DOI:** 10.1093/jisesa/ieu154

**Published:** 2014-01-01

**Authors:** Junhua Wang, Ling Zeng, Zhaojun Han

**Affiliations:** ^1^ Department of Entomology, Key Laboratory of Agriculture Ministry in College of Plant Protection, Nanjing Agricultural University, Nanjing 210095, China; ^2^ Department of Plant Protection, South China Agricultural University, Guangzhou 510642, China

**Keywords:** oriental fruit fly, geographic population, low-temperature stress, protective enzyme, cold-tolerant substance

## Abstract

The cold hardiness of larvae, pupae, and adults of the oriental fruit fly, *Bactrocera Dorsalis* (Hendel) (Diptera: Tephritidae) was characterized first, and then body water, total sugar and glycerol contents, and activities of superoxide dismutase (SOD), catalase (CAT), peroxidase (POD), and alcohol dehydrogenase (ADH) of different geographical populations subjected to suitable rearing conditions and under sublethal low-temperature stress were compared. The cold hardiness of different populations was well correlated with the latitudes of distributions. The northern marginal population (31.6° N) had higher cold tolerance than southern populations (23.1° N and 24.3° N). Among different life stages, larvae had the least cold tolerance, whereas pupae had the most tolerance. Under suitable rearing conditions, the marginal population had lower activities of all four tested enzymes than that of the southern populations and also had lower body water and higher total sugar and glycerol contents. The low-temperature stress induced higher SOD, CAT, POD, and ADH activities of all tested life stages and of all tested populations with higher increase intensity in adults and pupae than in larvae. The increase intensity was higher in the marginal population than in the southern populations. Pupae in the marginal population and adults in the southern populations showed the largest activity enhancement, which agreed with the insect’s overwinter stages in their respective locations. Lower temperature stress lowered body water and total sugar contents and increased glycerol contents. The results revealed a strong correlation between the cold hardiness of a population and the concentration or activity of various biochemicals and enzymes known to be involved in cold tolerance. The marginal population of *B. dorsalis* might have evolved a new biotype with better adaption to low temperature.

The oriental fruit fly, *Bactrocera dorsalis* (Hendel) (Diptera: Tephrididae), is a very destructive pest of fruit and vegetable with a very broad host range. The species is currently well established in tropical and subtropical regions of many South East Asia countries including China, Pacific islands, and Hawaii, causing great damage wherever it occurs ( Mau 2007 ). *B. dorsalis* has been frequently intercepted at ports around the world ( Stephens et al. 2007 ) and is characterized as chronically invading species with great potential to invade into new territory ( Mangan and Moreno 2002 ). A previous study suggested that the risk of *B. dorsalis* getting into temperate zones is low ( Stephens et al. 2007 ). However, in China, the presence of *B. dorsalis* has been documented gradually from south to north in Hainan, Guangdong, Guangxi, Fujian, Yunnan, Guizhou, Sichuan, and even Jiangsu provinces, and some of the areas have distinct seasons ( Ye 2001 , Ren et al. 2007 ). Characterizing and understanding the cold hardiness and mechanism(s) of this species are essential in predicting its potential distribution areas.

Ambient temperature is one of the important constrains to insect geographic distribution with great implications on behavior, physiology, and biochemistry of an insect. To combat unavoidable low temperature, insects can arm themselves with behavioral adaptations, such as long distance migration or vertical migration into the soil, and/or with adaptive physiological and biochemical processes such as reducing free body water, accumulating cold-tolerant substances (antifreezes), synthesizing ice-nucleating agent, or engaging rapid cold-hardening responses ( Lee and Denlinger 1991 , Li and Gong 1998 , Li et al. 2004 ). Many species have formed geographically distinct populations or biotypes with different cold-hardiness levels as they expand their distribution range ( Jing and Kang 2003 ). In recent years, there were reports of *B. dorsalis* invading into more northern areas and established persistent marginal populations significantly enough to cause damage in China. However, no apparent geographic variation in cold hardiness was found ( Ren et al. 2007 ).

As *B. dorsalis* extends its distribution to more temperate zones, enhancing tolerance to low temperature is one immediate task. Insect death due to low temperature can be caused by tissue freezing or by cold shock or other types of injury associated with low temperature rather than simple tissue freezing ( Bale 1996 , Zhao et al. 2010 ). Cold hardiness involves complex physiological processes, and characterizing cold-tolerant substances and protective enzyme activity under sublethal chill conditions is an important step in understanding this process. In this study, we first characterized the effect of low-temperature stress on the survival of larva, pupa, and adult using a population from a well-established southern zone (Fujian) to identify sublethal low-temperature conditions of each developmental stage. Then, the contents of body water, total sugar, glycerol, and the activity of four major protective enzyme systems in insects, superoxide dismutase (SOD), catalase (CAT), peroxidase (POD), and alcohol dehydrogenase (ADH) were compared under normal and sublethal chill conditions using three different geographic populations. The comparative studies provide information on cold hardiness variations of life stages of *B. dorsalis* and different geographical populations. Such information serves as a useful reference in predicting future geographic establishments, consequently benefiting the management of this troublesome insect.

## Materials and Methods

### 

#### Insects

Low-temperature stress tests were conducted in 2007 using a population (Fujian) collected from lychee ( *Litchi chinensis* Sonn) on the campus of Fujian Agriculture and Forestry University in Fujian province (26.0° N, 119.3° E). The comparative studies were conducted in 2008 using three populations collected from three geographic locations. Guangzhou population was collected from carambola ( *Averrhoa carambola* L.) in Yangtao park, Guangzhou (23.1° N, 113.3° E); Xiamen population was collected from common guava ( *Psidium guajava* L.) in Tongan orchard, Xiamen (24.3° N, 118.1° E); and Wuxi population was collected from oranges ( *Citrus reticulata* Blanco) in Binhuqu citrus orchard in Wuxi (31.6° N, 120.3° E, an invaded northern marginal population). Infested fruits were collected from the different locations and brought back to the insectaria laboratory of Nanjing Agricultural University. Adults emerged from the infested fruits were collected and identified and reared on minced fresh bananas. Flies of different growth stages were selected from the F3 generation for testing. The rearing conditions were 25 ± 0.5°C, 70–80% relative humidity (RH), and photoperiod 14:10 (L:D) h.

#### Low-Temperature Stress Test

The lethal effect of low temperature on different development stages was evaluated with combinations of five temperature settings (10, 5, 0, −5, and −10°C) and six treatment durations (1, 3, 6, 12, 24, and 48 h). Third-instar larvae, 3-d-old pupae, or 2-d-old adults (only four temperature settings) were subjected to different combinations of temperature and duration (treatments) in groups of 30 individuals placed in a Petri dish with minced fresh bananas as food source. There were three replications (Petri dish) for each treatment. After the treatments, the insects were recovered under the rearing conditions for 24 h before mortality was recorded. The fly was considered died when no movement of any part of the body was observed when touched with a fine brush.

#### Comparative Test of Larval Cold Hardiness of Geographic Populations

Only larvae were assayed as it is the growth stage that sufficient number of individuals could be obtained for testing. Based on the results of the low-temperature stress test, treatment combination of 5°C × 6 h was selected for this test as it produced 20–25% mortality for Fujian population, which enables a better evaluation. Third-instar larvae of the three different geographic populations were subjected to the treatment in groups of 30 individuals with three replicates. Insects of each population under normal rearing condition were as controls. After treatment period ended, the insects were allowed to recover for 24 h before mortality was recorded as described above.

#### Sublethal Low-Temperature Stress Treatment

Based on the results of the low-temperature stress test, treatments that produced 20–25% mortality for Fujian population were selected for eliciting low-temperature stress. Treatment combinations of 5°C × 6 h, 0°C × 12 h, and 5°C × 12 h were selected for third-instar larvae, 3-d-old-pupae, and 3-d-old adults, respectively. Insects of different stages from the 3 geographic populations were subjected to their perspective treatments in groups of 20 individuals. At the end of each treatment, the insects were allowed to recover for 2 h under the rearing conditions. Live individuals were then collected and immediately stored at −70°C for body water, total sugar and glycerol contents, and enzyme activity testing. Insects of each population under normal rearing condition were also tested for comparison.

#### Body Water, Total Sugar, and Glycerol Measures

Total body water was measured based on the weight difference of insects before and after oven dried at 60°C for 48 h. Water content was expressed as percent dry body weight.

To measure total sugar content, two insects were washed with stilled water and dried with tissue paper. After weighting, the insects were manually homogenized using a glass rob in 20 μl of distilled water and some fine quartz sand. The homogenate combined with 380 μl 10% trichloroacetic acid solution used to rinse the glass rob was centrifuged at 1,468 × *g* for 5 min at 20°C. The supernatant was transferred into a glass test tube. The pellet was extracted once more with 400 μl of 10% trichloroacetic acid solution. The supernatants were combined and brought to 1 ml. Total sugar was measured by traditional Anthrone colorimetry ( Yemm and Willis 1954 ).

Glycerol content was measured using modified Fletcher method ( Fletcher 1968 , Chen and Kang 2004 ). Two insects were pretreated and homogenized in the same way as sugar content sample. The homogenate combined with 480-μl distilled water used to rinse the glass rob was centrifuged at 1,468 × *g* for 10 min. The supernatant was transferred into a glass test tube. The pellet was extracted once more with 500 μl distilled water. The supernatants were combined for glycerol content determination.

All measurements had three biological replications.

#### Enzyme Activity Assays

Enzyme source was prepared by weighting 10 insects homogenized with prechilled 0.9% physiological saline to a concentration of 10% (w/v) following the protocol requirement of assay kits. The homogenate was centrifuged at 1,468 × *g* for 10 min. The supernatant was carefully collected (avoiding the fat layer) and used.

All four types of enzymes were determined using commercially available standard assay kits (Nanjing Jiancheng Science and Technology Company Ltd. Nanjing, China) by following the manual instructions. The kit for SOD activity (A001) was based on xanthine oxidase following the method of McCord and Fridovich (1969) . The reaction absorbance at 550 nm was measured by a visible spectrophotometer (Eppendorf, Bio Photometer AG 22331 Hamburg), and the activity was expressed as U/mg soluble protein. One unit of SOD was defined as the activity of enzyme that inhibited the oxidation by 50% that was determined from a predetermined inhibition curve.

CAT activity was measured using a kit (A007-2) based on the method described by Rao et al. (1996) with H _2_ O _2_ as a substrate. The absorbance of reaction mixtures was measured at 405 nm, and enzyme activity was calculated. One unit was defined as the activity of 1 mg of enzyme protein that catalyzed the reduction of hydrogen peroxide causing 0.5 OD difference per second.

The activity of POD was measured with a kit (A084-4) based on the method of Chance and Maehly (1955) . The absorbance of reaction mixtures was measured at 420 nm. One unit was defined as the activity of 1 mg of enzyme protein that catalyzed 1 μg of the substrate per minute of reaction.

ADH activity was determined using a kit (A083-1) based on the method of Hu and Liu (2001) with β-nicotinamide adenine dinucleotide hydrate as a substrate. The absorbance of reaction mixtures was measured at 340 nm and used for activity calculation. One unit was defined as the activity of 1 mg of enzyme protein that catalyzed 1 nmol of product per minute of reaction.

The protein content of the enzyme source was measured with the Bradford method and bovine serum albumin as the standard ( Bradford 1976 ).

#### Data Analysis

All data were processed using DPS 2000 software. One-way or two-way analysis of variance (ANOVA) was performed followed by Duncan test for mean comparison at α = 0.05 and also Holm–Bonferroni correction. Body water, total sugar, glycerol, and enzyme activity between treatment and control within each geographic population was compared by *t* -test (one tail).

## Results

### 

#### Response of Different Growth Stages to Low-Temperature Stress

The mortality caused by low-temperature stresses of different durations is listed in Table 1 . As expected, ANOVA analysis indicated that low temperature significantly increased mortality for all insect stages ( *P* < 0.001). Exposure duration also had significant effect ( *P* < 0.001) on the mortality regardless of insect stage. As the duration time increased, mortality increased significantly.

**Table 1.** Mortality of *B. dorsalis* caused by stress of different combinations of low temperature and duration *^a^*

Temperature(°C)	Treatment duration					
	1 h	3 h	6 h	12 h	24 h	48 h
Third-instar larvae						
10	4.97 ± 0.17	10.10 ± 0.31	15.97 ± 0.38	20.00 ± 0.46	25.93 ± 0.50	31.00 ± 0.35
5	10.03 ± 0.27	17.00 ± 0.30	21.17 ± 0.29	25.00 ± 0.52	31.97 ± 0.09	37.83 ± 0.18
0	25.00 ± 0.52	59.00 ± 0.21	87.07 ± 0.23	96.67 ± 0.03	98.90 ± 1.10	100.00
−5	36.97 ± 0.32	67.10 ± 0.40	89.17 ± 0.35	98.90 ± 1.10	100.00	100.00
−10	96.93 ± 0.03	100.00	100.00	100.00	100.00	100.00
Three-day-old pupae						
10	0	0	0	0	0	5
5	0	0	6.07 ± 0.33	10.07 ± 0.35	15.07 ± 0.18	17.00 ± 0.50
0	7.93 ± 0.39	13.03 ± 0.27	18.00 ± 0.10	23.10 ± 0.47	27.07 ± 0.50	31.13 ± 0.24
−5	17.10 ± 0.21	23.03 ± 0.18	23.97 ± 0.09	26.93 ± 0.38	47.97 ± 0.09	76.90 ± 0.47
−10	36.03 ± 0.20	45.10 ± 0.21	70.93 ± 0.24	84.97 ± 0.50	93.10 ± 0.66	100.00
Two-day-old adults						
10	0	0	0	0	13.90 ± 0.21	18.10 ± 0.10
5	2.23 ± 1.12	8.00 ± 0.17	17.00 ± 0.21	22.10 ± 0.38	23.97 ± 0.09	34.13 ± 0.42
0	6.07 ± 0.33	17.00 ± 0.30	27.07 ± 0.27	32.87 ± 0.43	47.07 ± 0.44	61.03 ± 0.24
−5	31.27 ± 0.64	62.03 ± 0.29	86.90 ± 0.38	96.63 ± 0.03	100.00	100.00

*^a^* Mean ± SD ( *n* = 3).

The larvae exhibited the lowest cold tolerance compared with pupae and adults, whereas pupae were most cold tolerant as they suffered relatively low mortality in all the treatments. For example, at 10°C × 48 h, the mortality of larvae, pupae, and adults was 31, 5, and 18%, respectively. At 0°C × 48 h, the corresponding mortality was 100, 31, and 61%.

#### Variation in Larval Response to Low-Temperature Stress of Geographic Populations

The response to low-temperature stress of different geographic populations was evaluated using third-instar larvae with the treatment of 5°C × 6 h. Wuxi population suffered the least mortality compared with Guangzhou and Xiamen populations, and Xiamen population had lower mortality than that of Guangzhou population ( Table 2 ). The differences in mortality indicated that cold tolerance of the larvae increased as the latitude of collection sites increased. Insects of each population under the normal rearing condition suffered no mortality.

**Table 2.** Mortality of third-instar larvae of *B. dorsalis* from different geographic populations under stress treatment at 5°C × 6 h

Populations	Mortality (%) *^a^*
Guangzhou (23.1° N)	34.4 ± 5.1 a
Xiamen (24.3° N)	26.7 ± 3.3 a
Wuxi (31.6° N)	14.4 ± 1.9 b
*F, P* values	22.439, 0.0016

*^a^* Mean ± SD ( *n* = 3) values follows different letter are significantly different (Holm–Bonferroni correction, *P* < 0.05/3).

#### Body Water, Total Sugar, and Glycerol Contents in Response to Low-Temperature Stress

Body water contents were significantly different among geographic populations where Wuxi population had significantly lower water contents than that of Guangzhou and Xiamen population regardless of life stages ( Table 3 ), even tested with Holm–Bonferroni correction. Within population, low-temperature stress significantly decreased body water contents for all three life stages ( *P* < 0.01).

**Table 3.** Body water, total sugar, and glycerol contents under control (normal) or low-temperature stress *^a^*

Populations	Guangzhou	Xiamen	Wuxi	*F, P* values
Water contents (%)				
Larva control	71.31 ± 0.38 a	70.95 ± 0.21 a	69.37 ± 0.38 b	29.65, **0.0008**
Larva stressed	70.19 ± 0.39 a	68.94 ± 0.20 a	68.20 ± 0.36 b	31.17, **0.0007**
Pupa control	70.84 ± 0.18 a	70.50 ± 0.03 b	70.07 ± 0.24 c	15.54, **0.0042**
Pupa stressed	69.70 ± 0.16 a	69.36 ± 0.04 a	68.63 ± 0.26 b	18.67, **0.0027**
Adult control	71.54 ± 0.09 a	71.23 ± 0.47 a	70.30 ± 0.17 b	14.58, **0.005**
Adult stressed	70.34 ± 0.10 a	70.03 ± 0.45 a	69.09 ± 0.16 b	15.75, **0.0041**
Total sugar (mg/g)				
Larva control	3.20 ± 0.013 b	3.21 ± 0.012 b	3.24 ± 0.006 a	13.48, **0.006**
Larva stressed	3.16 ± 0.014 b	3.17 ± 0.013 ab	3.20 ± 0.006 a	6.23, 0.034
Pupa control	3.21 ± 0.010 b	3.23 ± 0.009 b	3.27 ± 0.005 a	35.38, **0.0005**
Pupa stressed	3.17 ± 0.001 a	3.19 ± 0.008 b	3.22 ± 0.005 b	24.59, **0.0013**
Adult control	3.23 ± 0.012 b	3.23 ± 0.009 b	3.25 ± 0.007 a	7.18, 0.026
Adult stressed	3.18 ± 0.012	3.19 ± 0.010	3.20 ± 0.009	3.95, 0.080
Glycerol (mg/g)				
Larva control	4.90 ± 0.051	4.92 ± 0.060	4.98 ± 0.006	2.28, 0.184
Larva stressed	5.13 ± 0.036 b	5.15 ± 0.064 ab	5.25 ± 0.010 a	6.85, 0.028
Pupa control	4.95 ± 0.016 b	4.95 ± 0.007 b	5.08 ± 0.074 a	8.21, 0.019
Pupa stressed	5.19 ± 0.016 b	5.20 ± 0.016 b	5.38 ± 0.060 a	25.19, **0.0012**
Adult control	4.94 ± 0.014 b	4.95 ± 0.014 b	5.01 ± 0.026 a	10.76, **0.0104**
Adult stressed	5.21 ± 0.010 b	5.22 ± 0.009 b	5.29 ± 0.022 a	29.06, **0.0008**

*^a^* Mean ± SD ( *n* = 3) values follows different letter are significantly different (Duncan’s test, *P* < 0.05. The values in bold is still significant even corrected with Holm–Bonferroni method) within the same treatment among populations.

Wuxi population had a little higher sugar contents than that of Guangzhou and Xiamen populations, especially for the pupa stage. Both Duncan’s test and the test of Holm–Bonferroni method showed that it was significant. Low-temperature stress significantly decreased total sugar contents regardless of population or life stages ( *P* < 0.05).

The trend for glycerol content was different from body water. Significant difference among populations was observed for pupa or adult but not for larva. Wuxi population had higher glycerol compared with Guangzhou and Xiamen population. Comparisons ( *t* -test) between control and stressed indicated that low-temperature stress increased the glycerol for all life stages of all populations ( *P* < 0.05).

#### Comparisons of SOD Activities in Response to Low-Temperature Stress

The SOD activity in larvae, pupae, and adults of different geographic populations under normal rearing conditions (control) or low-temperature stress (treated) is listed in Table 4 . Adults generally had higher SOD activity followed by pupae and larvae. Under normal rearing conditions, Wuxi population had significantly lower SOD activity compared with Guangzhou population for all three tested life stages. Little difference between Guangzhou and Xiamen populations was observed. Low-temperature stress enhanced SOD activity obviously in the treated insects regardless of life stage and population, but the increase of activity due to the treatment was greater for Wuxi population compared with that of Guangzhou and Xiamen populations, and such increase was most prominent in pupae (overwinter stage). These results suggest that Wuxi (northern marginal) population had stronger response to low-temperature stress.

**Table 4.** Activity of SOD in different life stages of *B. dorsalis* of different geographic populations

Stages	Populations	Enzyme activity (U/mg protein) *^a^*	
		Control	Cold stressed
Third-instar larvae	Guangzhou	129.12 ± 5.65 a	148.82 ± 6.08
	Xiamen	124.33 ± 2.81 b	144.46 ± 2.84
	Wuxi	111.73 ± 3.30 b	140.92 ± 4.78
	*F* , *P* values	14.32 **0.0052**	2.08 0.21
Three-day-old pupae	Guangzhou	133.26 ± 3.26 a	157.25 ± 3.68 ab
	Xiamen	129.34 ± 1.31 a	154.08 ± 0.75 a
	Wuxi	119.50 ± 3.16 b	162.81 ± 4.48 b
	*F* , *P* values	20.27 **0.0021**	5.14 0.05
Two-day-old adults	Guangzhou	142.73 ± 4.72 a	172.28 ± 4.64
	Xiamen	139.59 ± 0.90 a	172.28 ± 0.90
	Wuxi	129.89 ± 2.03 b	174.93 ± 5.09
	*F* , *P* values	14.81 **0.0048**	0.44 0.67

*^a^* Mean ± SD ( *n* = 3) values within each insect stage and the same treatment (column) followed by different letters were significantly different among populations (Duncan’s test, *P* < 0.05. The values in bold are still significant even corrected with Holm–Bonferroni method), the same is below.

#### Comparisons of CAT Activities in Response to Low-Temperature Stress

In general, adults also had higher CAT activity followed by pupae and larvae ( Table 5 ). Under normal rearing conditions, Wuxi population had significantly lower CAT activity compared with Guangzhou and Xiamen populations for the three tested life stages, while no difference was found between Guangzhou and Xiamen populations. Under low-temperature stress, larval CAT activity of Wuxi population was still significantly lower than that of the other two populations, whereas adult and pupa CAT activity had no difference among populations. Comparing the control and treated insects within population, low-temperature stress significantly increased CAT activity in larvae, pupae and adults. Again Wuxi population had relatively higher response than the other two more southern populations, and pupae had relatively higher response than larvae and adults ( Table 5 ).

**Table 5.** Activity of CAT in different life stages of *B. dorsalis* of different geographic populations

Stages	Populations	Enzyme activity (U/mg protein)	
		Control	Cold stressed
Third-instar larvae	Guangzhou	24.45 ± 0.52 a	27.95 ± 0.65 a
	Xiamen	23.64 ± 0.46 a	27.11 ± 0.51 a
	Wuxi	20.75 ± 0.45 b	24.56 ± 0.60 b
	*F* , *P* values	50.40, **0.0002**	27.04, **0.001**
Three-day-old pupae	Guangzhou	26.46 ± 1.00 a	31.34 ± 1.05
	Xiamen	26.05 ± 0.49 a	31.21 ± 0.46
	Wuxi	24.01 ± 0.83 b	31.88 ± 0.90
	*F* , *P* values	8.11, **0.02**	0.53, 0.61
Two-day-old adults	Guangzhou	27.64 ± 0.90 a	34.50 ± 1.37
	Xiamen	26.70 ± 0.31 a	33.69 ± 0.45
	Wuxi	25.14 ± 0.71 b	32.49 ± 0.81
	*F* , *P* values	10.25, **0.012**	3.34, 0.11

#### Comparisons of POD Activities in Response to Low-Temperature Stress

Similarly, adults had the highest level of POD activity followed by pupae and larvae. Under normal rearing conditions, insects of Wuxi population had significantly lower POD activity than that of Guangzhou and Xiamen populations for all tested life stages, whereas a little difference was found between Guangzhou and Xiamen population only in larvae ( Table 6 ). Under low-temperature stress, the larvae of Wuxi population still had POD activity lower than that of Guangzhou population. However, the pupae of Wuxi population showed the highest POD activity, though Holm–Bonferroni correction found no significant difference. Similar to SOD and CAT, low-temperature stress significantly increased the POD activity across life stages with higher increase in Wuxi population ( Table 6 ).

**Table 6.** Activity of POD in different life stages of *B. dorsalis* of different geographic populations

Stages	Populations	Enzyme activity (U/mg protein)	
		Control	Cold stressed
Third-instar larvae	Guangzhou	181.10 ± 3.10 a	205.20 ± 4.13 a
	Xiamen	173.42 ± 2.20 b	197.61 ± 1.57 b
	Wuxi	156.30 ± 3.56 c	196.84 ± 3.90 b
	*F* , *P* values	53.51 **0.0001**	5.54, 0.043
Three-day-old pupae	Guangzhou	187.09 ± 3.84 a	216.09 ± 3.29 ab
	Xiamen	180.39 ± 2.74 a	210.82 ± 2.05 a
	Wuxi	162.85 ± 4.20 b	222.36 ± 5.53 b
	*F* , *P* values	35.36 **0.0005**	6.58, 0.031
Two-day-old adults	Guangzhou	196.63 ± 3.30 a	234.47 ± 4.87
	Xiamen	192.01 ± 3.60 a	231.56 ± 4.04
	Wuxi	167.95 ± 2.28 b	227.32 ± 1.6 2
	*F* , *P* values	73.53 **0.0001**	2.73, 0.14

#### Comparisons of ADH Activities in Response to Low-Temperature Stress

The ADH activity continued the trend as the three previously mentioned enzymes. Adults had the highest activity followed by pupae and larvae. Under normal rearing conditions, insects of Wuxi population had significantly lower activity compared with Guangzhou and Xiamen populations for all tested life stages, whereas a little difference was also found between Guangzhou and Xiamen population in larvae and adults ( Table 7 ). Under low-temperature stress, all populations had similar ADH activity in larvae and and pupae. However, adults of Wuxi population still had significant low activity. Obviously, low-temperature stress significantly increased the ADH activity across life stages with higher increase in the pupae and larvae of Wuxi population ( Table 7 ).

**Table 7.** Activity of ADH in different life stages of *B. dorsalis* of different geographic populations

Stages	Populations	Enzyme activity (U/mg protein)	
		Control	Cold stressed
Third-instar larvae	Guangzhou	73.5 ± 0.7 a	76.0 ± 1.0 a
	Xiamen	71.5 ± 0.5 b	74.3 ± 0.4 b
	Wuxi	68.2 ± 0.9 c	74.2 ± 0.6 b
	*F* , *P* values	43.30, **0.0003**	5.63, 0.042
Three-day-old pupae	Guangzhou	80.2 ± 1.4a	83.8 ± 1.2 ab
	Xiamen	78.6 ± 0.9 a	83.0 ± 1.2 a
	Wuxi	73.3 ± 0.6b	85.8 ± 0.7 b
	*F* , *P* values	39.01, **0.0004**	5.53, 0.044
Two-day-old adults	Guangzhou	87.3 ± 1.2 a	94.6 ± 1.0 a
	Xiamen	84.4 ± 0.5 b	92.1 ± 0.6 b
	Wuxi	74.6 ± 0.8c	84.8 ± 1.2 c
	*F* , *P* values	181.61, **0.0001**	79.33, **0.0001**

## Discussion

As many insects do, *B. dorsalis* typically has different phenology that depends on climate conditions and geographical features ( Ye 2001 ). Our study demonstrated that cold tolerance of the different geographic populations was well correlated with the latitudes of their collection sites, e.g., the mortality of third-instar larvae after the treatments of 5°C for 6 h was 34.4, 26.7, 21.2, and 14.4% for Guangzhou (23.1° N), Xiamen (24.3° N), Fujian (26.0° N), and Wuxi (31.6° N) populations, respectively. This result implies that *B. dorsalis* adapts cold climate gradually during expanding its distribution northern ward in China. However, when it invaded and colonized in more temperate zone Wuxi, this fly varied dramatically in its occurrence. In Guangzhou area, the fly is active year round with seven generations, overwinter with dormant adults in orchards ( Liang et al. 1993 , Wu et al. 2007 ). Multiple generations (5–6) also occur in Xiamen and Fuzhou area. Adults survive the winter by hiding on the host trees or in tall grasses and actively forage for food during warmer days. Adults could die during the winter if no food is available, and the winter low temperature is lethal to eggs, larvae, and pupae ( Zhang et al. 1998 ). The area south of Yangtze River (31.30∼34.59° N) has been regarded as only potential zone of *B. dorsalis* colonization, and in recent years, it was confirmed that this species has established stable populations in this area ( Luo et al. 2009 ). In this more temperate zone, the flies overwinter as pupae in ∼5 cm deep soil and have apparent diapause in spring time, thus no adult activity can be detected during the period from December to May of the following year ( Luo et al. 2009 ).

The low-temperature stress tests of this study revealed that pupae were the most cold tolerant followed by adults and then larvae. These findings agree with the phenology of the flies at different sites, where adults are the overwinter stage in warmer areas, and pupae are the overwinter stage at colder areas. Otherwise, this study also revealed the northern marginal population (Wuxi population) is significantly different from the two southern populations (Guangzhou and Xiamen) in biochemistry and physiology.

Enzymes are biological molecules responsible for catalyzing numerous reactions that are essential to life. SOD, CAT, and POD are three important types of enzymes in organisms. Together they play crucial roles in cellular detoxification of damaging superoxide radicals typically formed under stress conditions such as extreme heat and cold ( Lozinskaya et al. 2004 , Zhang et al. 2006 , Feng et al. 2008 ). SOD catalyzes the dismutation of superoxide radical into hydrogen peroxide (H _2_ O _2_ ), which is subsequently removed by CAT and POD into water and oxygen ( Fridovich 1977 ). ADH is a group of dehydrogenase enzymes involving in breaking down toxic alcohols in animals and many other important physiological processes including the production and metabolism of cold-tolerant chemicals ( Holden and Storey 1994 ). Our comparative enzyme activity study revealed that these four enzyme systems were very much relevant to cold hardiness of *B. dorsalis* because the activity corresponded well with cold hardiness, i.e., higher enzyme activity in pupae and adults with lower mortality under cold stresses when compared with larvae. Similar correlation was identified in a cold tolerance study with leaves of evergreen woody plants ( Chen et al. 2006 ). The results also revealed that the effect of low-temperature stress on SOD, CAT, POD, and ADH was similar, i.e., higher activity under stress condition. Furthermore, such increase of enzyme activity was more intense in more cold tolerant life stage (pupae and adults) and in more northern populations. The demonstrated enzyme activity responses to the low-temperature treatment agree with the different roles played by those enzymes in living organisms and suggest that these four protective enzyme systems play a role in cold tolerance of *B. dorsalis* .

The apparent physiology difference of the northern marginal population was reflected by lower body water content, higher sugar and glycerol content, and lower enzyme activity than that of Guangzhou and Xiamen under suitable temperature condition and more dramatic responses under low-temperature stress. In addition, the greatest enzymatic response to low temperature was found in pupae of Wuxi population. The low enzyme activity under suitable condition implied that the flies in northern marginal population had changed and decreased their enzyme demand. The dramatic responses of the enzymes under low-temperature stress mean more enzymes for maintaining cold hardness. Guangzhou and Xiamen are geographically close with similar climatic conditions. The body water, total sugar, glycerol contents, and protective enzyme activity of the populations from those two locations was also similar. Similarly, Ren et al. (2007) reported that no differences in cold hardiness among *B. dorsalis* populations from Fuzhou, Haikou, Nanning, Guangzhou, and Yuanjiang.

Cold-tolerant substances and enzyme activity in organisms are controlled by genes, number of gene copies, and their expression. The findings in this study suggest that a permanent modification of cold hardiness control in Wuxi population may have occurred. Combined with the fact that populations in northern marginal distribution zones overwinter as pupae rather than adults for populations in the south and the overwinter pupae have an apparent diapauses period ( Wu et al. 2007 , Luo et al. 2009 ), we could tentatively conclude that the Wuxi (northern) populations are a different biotype from the southern populations. However, further delineation studies on the roles of the defensive enzymes in cold tolerance are needed.
